# Life Satisfaction, Positive Affect, and Sleep Impairment in Masters Athletes: Modulation by Age, Sex, and Exercise Type

**DOI:** 10.3389/fphys.2021.634433

**Published:** 2021-03-04

**Authors:** Savannah V. Wooten, Uwe Mittag, José Ramón Alvero Cruz, Sten Stray-Gundersen, Fabian Hoffmann, Sarah Michély, Stefan Möstl, Wolfram Sies, Edwin Mulder, Philipp Rauschendorfer, Philip D. Chilibeck, Jörn Rittweger, Hirofumi Tanaka

**Affiliations:** ^1^Department of Kinesiology and Health Education, The University of Texas at Austin, Austin, TX, United States; ^2^German Aerospace Center, Institute of Aerospace Medicine, Cologne, Germany; ^3^Department of Human Physiology and Sports Physical Education, University of Málaga, Málaga, Spain; ^4^Department of Cardiology, University Hospital Cologne, Cologne, Germany; ^5^College of Kinesiology, University of Saskatchewan, Saskatoon, SK, Canada; ^6^Department of Pediatrics and Adolescent Medicine, University of Cologne, Cologne, Germany

**Keywords:** veteran athlete, quality of life, mood, sleep quality, aging

## Abstract

**Introduction:**

The masters athlete has been proposed as a model of successful aging. Research studies investigating psychological outlook in older athletes have primarily addressed negative affects including depression, anxiety, and stress. The impact of lifelong exercise on positive affect and life satisfaction as well as sleep impairment that could impact on these psychological states is largely unknown.

**Methods:**

A series of questionnaires (general life satisfaction, positive affect, and sleep-related impairment) were administered to 240 masters athletes participating in the World Masters Athletics Championships. Total raw scores were converted into *T* scores for comparison with the general population. Meaningful difference was defined by the PROMIS^®^ as one-half standard deviation from the centering sample.

**Results:**

Meaningful differences were observed for improved general life satisfaction and reduced sleep impairment for all masters athletes. Positive affect did not reach the meaningful difference threshold. No significant sex differences were found for any of the questionnaires (all *p* > 0.05). Similarly, no significant differences were found between endurance, sprint, and strength/power sports for general life satisfaction (*p* = 0.18), positive affect (*p* = 0.46), and sleep impairment (*p* = 0.77). In general, life satisfaction increased with age (*r* = 0.15, *p* = 0.02), and sleep impairment trended towards reduction with age (*r* = −0.13, *p* = 0.05). Positive affect demonstrated no correlation with age (*r* = 0.09, *p* = 0.18).

**Conclusion:**

This study demonstrates that the lifestyles of masters athletes contribute to improved general life satisfaction and reduced sleep impairment but not improved positive affect. The beneficial effects were observed irrespective of age, gender, and sporting types.

## Introduction

The world’s aging population has been increasing rapidly particularly in industrialized countries. Population aging will inevitably affect healthcare systems and individual perspectives on planning for longer and healthier lives (Lutz et al., 2008). Moreover, an ongoing objective of aging research is to identify strategies which increase health span and reduce age-associated morbidities (Fnnzs, 1980). Successful aging has been deemed a multi-faceted domain consisting of physical, psychological, cognitive, and social function aspects (Geard et al., 2020). When compared with sedentary, age-matched peers, masters athletes are able to delay age-related decrements in physical function and independence and compress morbidity (Hawkins et al., 2003; Lazarus and Harridge, 2017).

The masters athlete has been proposed as a model of successful aging and is thought to demonstrate high physiological reserve, intrinsic motivation to succeed, and positive psychological outlook due to incorporation of chronic exercise over the lifespan (Shephard et al., 1995; Hawkins et al., 2003; Tanaka and Seals, 2003; Tanaka et al., 2011). Research investigating psychological outlook in older athletes has primarily addressed negative affects including depression, anxiety, and stress (Heo and Lee, 2010; Bardhoshi et al., 2016). Masters athletes have been previously reported to have significantly lower depression, anxiety, and stress scale—21 scores compared with nonclinical normative data populations (Bardhoshi et al., 2016). However, physical activity has long been established to provide vast physiological, mental, and psychosocial benefits for aging populations outside of the negative domains (Taylor et al., 1985; Spirduso and Cronin, 2001; Uchida et al., 2012; Oh et al., 2017). The impact of lifelong exercises on positive affect and life satisfaction is largely unknown. This is unfortunate because high life satisfaction is associated with decreased risk for mortality and predicts lower risk of all-cause and natural-cause morality (Xu and Roberts, 2010; Diener and Chan, 2011).

Positive affect is commonly associated with feelings of happiness, joy, excitement, enthusiasm, and contentment (Diener and Chan, 2011). Most importantly, individuals who demonstrate high positive affect delay onset of frailty and have increased functional independence and survival (Ostir et al., 2000, 2004). The beneficial effects of lifelong exercise on functional independence might be mediated by its impact on positive affect. However, this possibility has thus far not been addressed. Sleep is essential for both physiological functioning and psychological health (Lee et al., 2009) and has been receiving much less attention in research effort. Older adults commonly report greater sleep disturbance and insomnia, which contribute to impaired daytime function, reduced quality of life, and increased prevalence of psychiatric disorders (Ancoli-Israel and Roth, 1999; Lee et al., 2009). Little is known about the impact of lifelong physical activity on sleep impairment in masters athletes who regularly engage in strenuous exercises and compete in athletic competitions.

Accordingly, the overall aim of the present study was to investigate whether masters athletes demonstrate greater life satisfaction and positive affect and reduced sleep impairment, three domains that have been understudied and under-researched in masters athletes. Our working hypothesis was that masters athletes would exhibit more favorable levels in all three domains compared with the general population irrespective of age, sex, and sports discipline.

## Materials and Methods

The Masters Athletic Field Study 2018 (MAFS-18) was constructed to evaluate physical and mental domains of successful aging as revealed in masters athletes. The present data set is a subset of larger MAFS-18 study that assessed physical, mental, and clinical constituents of overall fitness. It was implemented during the 23rd World Masters Athletics Championships held in Málaga, Spain, between the 4th and 15th of September 2018, where 8,189 athletes registered for competitions. Data collection took place in the Málaga City Stadium in close proximity to the registration office. The study was approved by the Masters Athletics organizing committee, and the research protocol was reviewed and approved by the ethical commission of the North Rhine Medical Association (Ärztekammer Nordrhein lfd Nr 2018171). The study has been registered with the German register for clinical trials^1^ with identifier DRKS00015172 before study commencement. The only inclusion criterion was that participants competed in the championships, and injuries that interfered with the testing procedures were criteria for exclusion.

### Participants

Initially, the MAFS-18 study had been designed with a sample size of 120 participants. However, overwhelming interest on the side of the athletes allowed us to include a total of 256 masters athletes. Of these, 240 athletes aged between 35 and 91 years (mean 58 ± 12 years) participated in the present sub-study (Table 1). All the participants provided their written informed consents.

**TABLE 1:** Participant characteristics.

	**All masters athletes**	**Endurance**	**Sprint**	**Strength/power**
Male/female (*n*)	155/85	56/37	69/25	30/23
Age (years)	58 ± 12	58 ± 11	56 ± 12	60 ± 13
Height (cm)	170.3 ± 8.9	167.5 ± 8.0	170.3 ± 7.7	174.1 ± 10.7
Body weight (kg)	69.7 ± 12.3	63.9 ± 9.3	70.6 ± 9.3	78.4 ± 15.8
BMI (kg/m^2^)	23.9 ± 3.1	22.7 ± 2.7	24.1 ± 2.2	25.8 ± 4.1
**Smoking (*n*)**				
Current	2	2	0	0
None	238	91	94	53
Former	44	18	15	11
Exercise training (h/week)	8.2 ± 4.8	8.9 ± 5.9	8.1 ± 4.1	7.4 ± 3.6
**Race/ethnicity (*n*)**				
White	164	61	61	42
Hispanic	36	17	13	6
Black	8	3	4	1
Afro-Caribbean	3	1	1	1
Asian	5	0	3	2
Not reported or others	24	11	12	1

*Data are means ± SD or *n*.*

### Protocol

Height and body weight were measured with a stadiometer and balance scale (MPE250K100HM, Kern und Sohn, Balingen, Germany). Training habits and general health were collected via self-administered electronic questionnaire with researcher present to assist with clarifications and questions. Three questionnaires developed by the National Institute of Health Patient-Reported Outcomes Measurement Information System (PROMIS^®^) were self-administered electronically onsite. PROMIS is a set of person-centered measures that were created by the NIH Common Fund and validated to be psychometrically reliable and precise (Cella et al., 2010; Rothrock et al., 2020). The questionnaires comprised (1) General Life Satisfaction–short form 5a, (2) Positive Affect–short form15a, and (3) Sleep-Related Impairment–short form 8a. General Life Satisfaction is a five-item questionnaire that deals with one’s cognitive evaluation of life experiences and whether one likes his/her life or not. Positive Affect is a 15-item questionnaire that evaluates feelings and mood that reflect a level of pleasurable engagement with their environment such as happiness, joy, excitement, enthusiasm, and contentment. Sleep-Related Impairment is an eight-item questionnaire that assesses perceived functional impairments during wakefulness associated with sleep problems, impaired alertness, sleepiness, and tiredness. These questionnaires were available in five different languages (English, Spanish, French, German, and Russian) to accommodate athletes from various parts of the world. The questionnaires were available in electronic form using web-based data capturing software REDCap (Harris et al., 2009, 2019).

### Statistical Analyses

Total raw score from the questionnaires were individually transformed into *T* scores using conversion charts from the PROMIS^®^ database (Driban et al., 2015; Rothrock et al., 2020). *T* score conversions have been validated and strongly correlate with participants’ actual responses to PROMIS short form items (*r* = 0.762–0.950) (Rothrock et al., 2020). Athletes were arbitrarily grouped into three sub-disciplines based on their self-rated best athletic competition events. The groupings consisted of the following sports: endurance (800 m run, 1,000 m run, 1,500 m run, 5 km run, 10 km run, marathon, steeplechase, track walk, and road walk), sprint (100 m run, 200 m run, 400 m run, hurdles, and triple jump), and strength/power (pole vault, shot put, weight throw, hammer throw, javelin throw, high jump, long jump, decathlon, heptathlon, throw pentathlon, and weight pentathlon). Some athletic events could arguably fall into different sub-disciplines (e.g., long jump into sprint, 400 m run to endurance). However, moving these events to different sub-disciplines did not influence our overall results. For all questionnaire score comparisons, the general United States population (pooled) from the 2000 US General Census (mean *T* score of 50 ± 10) was used as the centering sample for comparison to masters athletes (Fischer et al., 2018). Meaningful difference was defined by the PROMIS^®^ as one half standard deviation from the centering sample, which is five points from the reference population (Yost et al., 2011; Chen et al., 2018). One-way ANOVA was used to examine differences between sub-group means for sex, age, and sport. Pearson correlational and regression analyses were performed to evaluate associations of interest. Statistical significance was set at *p* < 0.05. The raw data supporting the conclusions of this article will be made available by the authors, without undue reservation.

## Results

**FIGURE 1:**
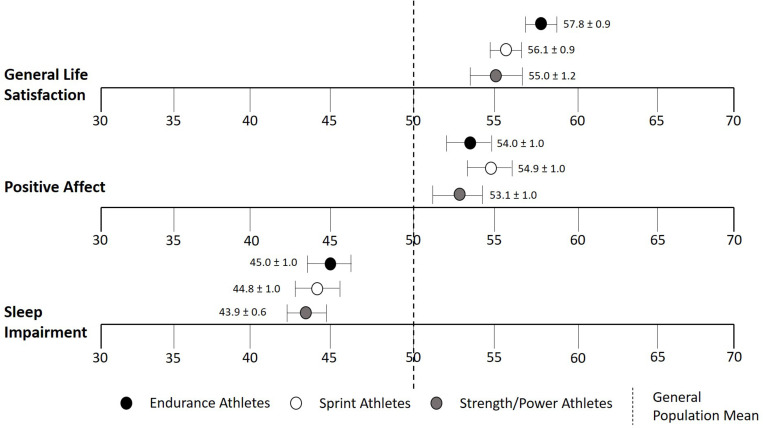
*T* scores of general life satisfaction, positive affect, and sleep impairment stratified by sport-discipline (endurance, sprint, and strength/power events). Centering sample (i.e., general population mean of 50) is shown as a dotted line. Data are means ± SEM.

Masters athletes divided into three sub-disciplines of sports groups including endurance, sprint, and strength/power events were well-matched for age, body mass index (BMI), exercise training volume, and demographic characteristics (Table 1). When comparing masters athletes with the general United States population, a meaningful difference was found for all three sport groups for general life satisfaction and sleep impairment but not for positive affect (Figure 1). There were no sports group-related differences in general life satisfaction (*p* = 0.18), positive affect (*p* = 0.46), and sleep impairment (*p* = 0.77).

**FIGURE 2:**
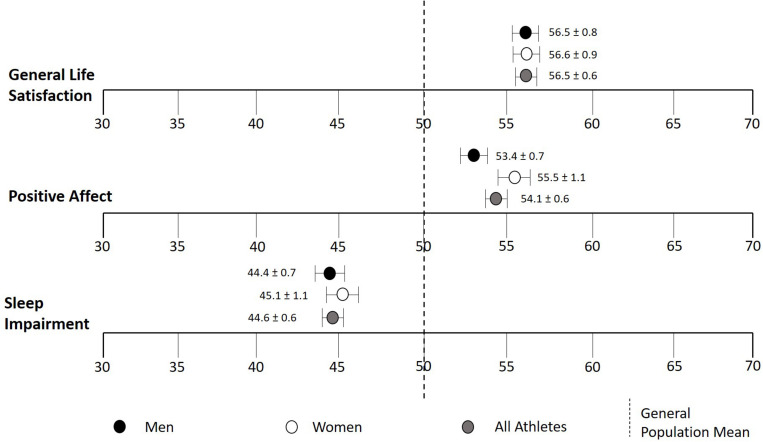
*T* scores of general life satisfaction, positive affect, and sleep impairment stratified by sex. Centering sample (i.e., general population mean of 50) is shown in a dotted line. Data are means ± SEM.

As shown in Figure 2, there were no significant differences in *T* scores between male and female masters athletes in general life satisfaction (*p* = 0.95), positive affect (*p* = 0.072), and sleep impairment (*p* = 0.57). Even when masters athletes were divided into men and women within sub-disciplines of sports, no significant gender/sex differences were found (*p* > 0.05; data not shown). However, women reached minimally important differences (MID) with an average of 5.5 points above the general population mean of 50 for positive affect, whereas men did not (3.4 points above the general population mean).

**FIGURE 3:**
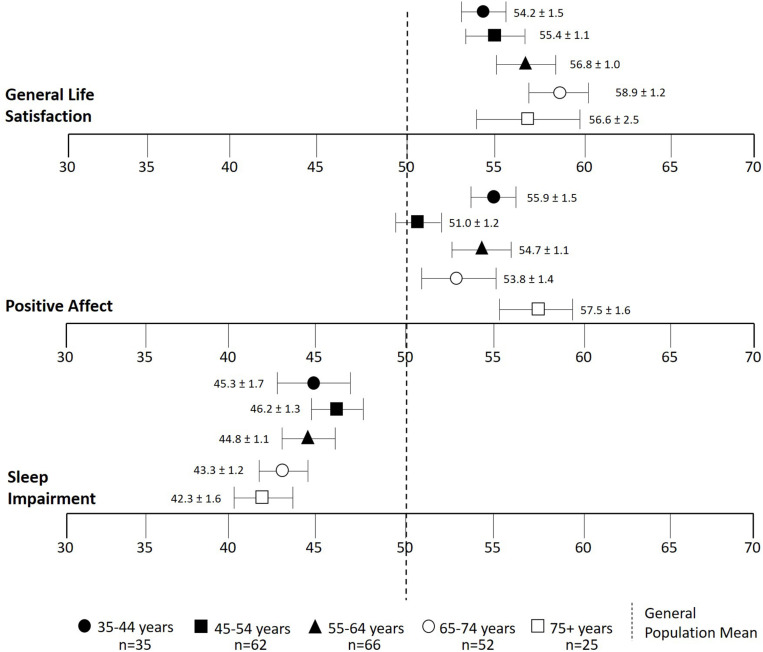
*T* scores of general life satisfaction, positive affect, and sleep impairment stratified by age groups. Centering sample (i.e., general population mean of 50) is shown in a dotted line. Data are means ± SEM.

There was a tendency for general life satisfaction to be greater with increasing age groups with the highest rating in the 65–74 age group (Figure 3). The 75+ age group reported the highest values for positive affect, but there was no apparent trend in association with age. Sleep impairment score had a tendency to decrease with increasing age groups with the lowest rating reported in the 75+ age groups.

When associations of interest were evaluated by correlation analysis, age was positively associated with general life satisfaction (*r* = 0.15, *p* = 0.02) and trended towards a negative association with sleep impairment (*r* = −0.13, *p* = 0.05). No association was found between age and positive affect (*r* = 0.09, *p* = 0.18). General life satisfaction was positively associated with positive affect (*r* = 0.54, *p* < 0.01) and negatively with sleep impairment (*r* = −0.31, *p* < 0.01). There was an inverse association between positive affect and sleep impairment (*r* = −0.40, *p* < 0.01). Training volume was not found to be associated with general life satisfaction (*r* = −0.03, *p* = 0.64), positive affect (*r* = 0.06, *p* = 0.42), nor sleep impairment (*r* = −0.04, *p* = 0.53).

## Discussion

The present study is the first to report positive affect and sleep impairment in a large cohort of international masters athletes. We found that masters athletes reported greater general life satisfaction and lower sleep impairment that are meaningfully different from the general population. More importantly, this was observed in both men and women, in athletes in different sporting disciplines, and throughout age groups. In contrast to our working hypothesis, meaningful difference was not detected for positive affect between masters athletes and the general population. The present study demonstrates that the physically active lifestyle of masters athletes can contribute to improved overall life satisfaction and reduced sleep impairment.

Life satisfaction is an important element for successful aging as it relates to the enhanced quality of life and longevity (Xu and Roberts, 2010; Diener and Chan, 2011). Compared with the general population, masters athletes reported greater life satisfaction regardless of age, gender, or sport disciplines. Age was positively associated with life satisfaction in masters athletes. These findings are consistent with previous observations in general populations (Baird et al., 2010; Gwozdz and Sousa-Poza, 2010), and the present study extends this trend to masters athletes. Studies in the general population found that self-reported life satisfaction was seen to steadily increase from young to old ages followed by a sharp decline in life satisfaction for the oldest individuals. The reduction in general life satisfaction among the oldest groups was often attributed to low levels of perceived health rather than objective health status (Gwozdz and Sousa-Poza, 2010). The age-associated trend was very similar in Masters athletes in the present study. A previous report on masters athletes found that the majority of respondents regarded their quality of life to be much better than that of their sedentary peers (Shephard et al., 1995). The present study offers support behind the notion that a physically active lifestyle confers increased general life satisfaction. Furthermore, a lack of differences in general life satisfaction between sub-disciplines of sports could be surprising as aerobic exercise is often perceived to provide greater overall health benefits. However, exercisers with various training routines (endurance, resistance, stretching, or combination) have reported similarly high quality of life (Oh et al., 2017).

Positive affect is associated with a number of critical elements to be successful in Masters athletics. Elevated motivation, commitment, and athletic performance have all been linked to positive affect, at least in young athletes (McCarthy et al., 2010). Goal settings that many Masters athletes engage in are also known to enhance positive affect (Wiese, 2007). Moreover, social-cognitive theory postulates that perception of enhanced or superior capabilities leads to increases in positive affect and that mastery in disciplines has been identified as one possible mechanism that could explain the exercise–mental health relationship (Bandura, 1986). Based on these collective observations, we hypothesized positive affect to be substantially greater in masters athletes. However, levels of positive affect did not reach meaningful difference thresholds in most masters athletes with the clear exception of those who were the oldest age groups (75+ years).

Although positive affect and life satisfaction are known to be positively correlated with one another as seen in the present study, they are two different components of well-being (De Neve and Oswald, 2012). General life satisfaction refers to a longer-term evaluation of one’s life, whereas positive affect is influenced by the short-term emotional experience (De Neve and Oswald, 2012). In this context, the gender difference seen within the masters athlete cohort is noteworthy. Women reached MID with an average of 5.5 points above the general population mean of 50. However, this was not significantly different from the average response of men (3.4 points above the general population mean). Women’s prescribed gender roles in society are more likely than men’s to confer attitudes and skills which resonate with emotional responsiveness and positive affect (Wood et al., 1989). The present study demonstrates that masters athletes are not exempt from this general gender bias.

Sleep impairment is common in older adults. In fact, quality of sleep decreases with advancing age (Espiritu, 2008). This condition contributes to reduced well-being and overall health of aging individuals (Borglin et al., 2005). Individuals who regularly exercise are known to have greater sleep time and sleep efficiency than individuals who do not exercise (Kredlow et al., 2015). This was traditionally thought to serve as the restoration of energy and bodily functions to recover from exercise bouts. However, this trend has not been universally observed as strenuous exercise appears to impair sleep (Driver et al., 1994). In fact, a meta-analysis has concluded that regular exercise has small beneficial effects on total sleep time and sleep efficiency (Kredlow et al., 2015). In the present study, masters athletes reported lower sleep impairments than the general population. This particular finding is consistent with a previous survey showing that 88% of masters athletes reported sleeping well or very well (Shephard et al., 1995).

One novel aspect of the present study is the evaluation of age, sex, and exercise/sport types as these factors appear to be significant moderators of sleep in a previous meta-analysis (Kredlow et al., 2015). We found lower sleep impairment irrespective of age, sex, and exercise types. Collectively, these results indicate that lifelong exercise would provide wide-reaching benefits on sleep. While the present study evaluates age, sex, and exercise/sport time, many additional factors play a role on quality and quantity of sleep and should be investigated in the future. Chronotypes, also known as the expression of circadian rhythmicity in an individual, and time of training (morning or evening) can have a large impact on psychophysiological performance and vice versa (Vitale and Weydahl, 2017). For example, individuals who are categorized as morning types among three distinct chronotype categories have been reported to have lower rate of perceived exertion and greater athletic performance in the morning than evening types or neither types (Vitale and Weydahl, 2017). Moreover, the presence or absence of family and work life as well as socioeconomic status can have a critical impact on sleep and general life satisfaction (Meltzer and Mindell, 2007; Maume et al., 2010; Loft and Cameron, 2014; Myllyntausta et al., 2018; Kim and Cho, 2020). Women, specifically, are known to have greater sleep debt due to daily employment and familial obligations (Maume et al., 2010). Contralaterally, individuals who transition from full-time work to statutory retirement experience reductions in sleep difficulties (Myllyntausta et al., 2018). General life satisfaction and positive affect were both found to be negatively associated with sleep impairment. General life satisfaction and positive affect have the capability of directly impacting sleep quality (Ong et al., 2017; Ness and Saksvik-Lehouillier, 2018). The reverse also stands true that sleep impairment can also affect life satisfaction and positive affect (Fairholme and Manber, 2015; Piper, 2016). These three disciplines examined in the present study co-exist in an interwoven matrix of continuous cause and effect. With respect to masters athletes, the present study demonstrates that a physically active lifestyle is able to positively mediate these three factors.

Clearly, more research is needed to elucidate the physiological mechanism underlying the impact of regular exercise on sleep. Chronic exercise improves sleep duration and quality through a series of proposed physiological mechanisms (Uchida et al., 2012). Repeated acute bouts of exercise create subsequent chronic effects such as reductions in blood glucose, release of growth hormone, and increased brain-derived neurotrophic factor production, all of which have been proposed to impact sleep both directly and indirectly (Robin, Travis et al., 1959; Takahashi et al., 1968; Morselli et al., 2012; Uchida et al., 2012; Schmitt et al., 2016). Moreover, improvements in fitness level, body composition, and heart rate/heart rate variability associated with chronic exercise habits may additionally influence sleep (Knutson and Van Cauter, 2008; Stein and Pu, 2012). Sleeps contributes heavily to neuronal plasticity and memory processes (Blissitt, 2001). Most importantly, when neglected, sleep impairment can cause deleterious effects that negatively impact learning, memory, quality of life, and depression.

### Limitations

Our study had several limitations that should be emphasized. In clinical settings, PROMIS questionnaire analysis commonly reports MID, which define a set minimum number of points between two group means to identify meaningful differences between questionnaire responses and correspond to meaningful change in an individual’s life. In the case of the present study, MID scores of masters athletes were not able to be compared to reference or subcategorized population norms (i.e., regularly active adults, sedentary adults, age- and sex-matched). The original NIH PROMIS norming sample was not powered to develop subgroup normative values and most importantly were not available for the questionnaires selected for study use. Future studies should seek to compare the present findings with control groups which can be sub-categorized by activity level, sex, and age. A meaningful difference in questionnaire responses in the present study was defined as 5 points from the reference population and was based on PROMIS questionnaires, which reported MID *T* scores ranging from 2 to 6 points from the general United States population mean *T* score of 50 (Yost et al., 2011; Chen et al., 2018). An additional limitation was the use of the general United States population as the centering sample. Given the European location of the international field study, the study incorporated predominantly European participants and fewer participants from North and South America, Africa, Australia, and Asia. Moreover, it can be assumed that these participants were from a variety of social and economic backgrounds. However, it has been previously established that US PROMIS scores are largely comparable with those obtained in the United Kingdom, France, and Germany (Fischer et al., 2018). No significant difference was detected between United States participants and participants from other countries for general life satisfaction nor positive affect. Given the self-reported, qualitative nature of the questionnaires, it must be noted that the competition environment, warm weather conditions, and performance may also have played a factor in the athlete’s responses. Although the sleep impairment questionnaire was intended to quantify chronic sleep impairment, jet lag that the athletes experienced at the international competition may have played a role in some of the participant responses. Sleep impairment was found to be lower among individuals who lived within 3 h of the competition location than individuals who live 4 h east (*p* = 0.02) and 4 h west (*p* = 0.02).

Finally, although the sample size of 240 may seem large in comparison with other published work in masters athletics, it amounts to only 3% of 8,189 athletes who participated in the Málaga Masters Athletics championships. Thus, it is possible that self-selection bias led to predominant participation of those with higher life satisfaction. Moreover, one could imagine that filling a questionnaire on life satisfaction in the setting of an athletics world championship may have led to somewhat higher ratings of life satisfaction in masters athletes, given how vigorously and how long many athletes had prepared for the international competition. A large fraction of athletes were still likely jet-lagged when they filled out the sleep questionnaires. It is plausible that the scores of sleep impairments may have been exaggerated. Therefore, future studies should attempt to involve even larger samples sizes, and data ought to be assessed at several times during the competition season.

## Conclusion

This study supports the claim that masters athletes have greater life satisfaction and reduced sleep impairment compared with the general United States population. Habitual exercise is the foundation of the physically active lifestyle of masters athletes and is the primary influence on both physiological reserve and quality of life which can ultimately contribute to healthy aging.

## Data Availability Statement

The raw data supporting the conclusions of this article will be made available by the authors, without undue reservation.

## Ethics Statement

The studies involving human participants were reviewed and approved by German Aerospace Center (DLR). The patients/participants provided their written informed consent to participate in this study.

## Author Contributions

All authors contributed to the study concept and design, data collection, and preparation of the manuscript.

## Conflict of Interest

The authors declare that the research was conducted in the absence of any commercial or financial relationships that could be construed as a potential conflict of interest.

## Footnotes

1 www.drks.de
